# Attitudes and Experience of Autism and Learning disability(LD): A Survey of Mental Healthcare Staff

**DOI:** 10.1192/bjo.2022.323

**Published:** 2022-06-20

**Authors:** Ogba Onwuchekwa, Conor Davidson, Vishal Sharma

**Affiliations:** Leeds & York Partnership NHS Foundation Trust, Leeds, United Kingdom

## Abstract

**Aims:**

To establish a baseline of staff experience and confidence in autism/LD. To inform how we deliver training going forward. To collect good practice examples of reasonable adjustments. To ascertain knowledge about the appropriate recording for information related to Autism/LD

**Methods:**

All clinical and non-clinical staff of Leeds &York Partnership Foundation Trust(LYPFT), Bradford District Care Foundation Trust(BDCFT), South West Yorkshire Foundation Trust(SWYFT), Voluntary sectors, Local authority and Leeds Community Health Care NHS Trust (LCH)were invited to take part in the anonymised “Staff Autism and LD Survey” through the various trust wide email bulletins. Smart Survey was the platform used. It took about 5–7 minutes to complete, and the survey period was from 21/09/21 to 01/11/21

**Results:**

A total of 225 members of staff across six organisations took part in the survey.

76% (170) were from LYPFT, 16(7%) from Voluntary Sector Organisations, 6%(14) from Local Authority and 3% from LCH 3%(7), Missing 14(6%), BDCFT 1%(2), SWYPFT 1%(2)

The majority were nurses 23% (52), followed by psychologists 10% (22).

18% (41) stated they would be interested in becoming an autism champion for their team/service.

Although 89% (200) had heard of the term “reasonable adjustments”, 36% (81) had never seen a ‘hospital passport’ for an autistic or learning-disabled service user.

Only 24 (11%) said they knew where to record reasonable adjustments on the electronic patient record

In general staff were marginally more confident in making reasonable adjustments for people with autism than those with LD

Majority of staff preferred : face-to-face training, followed by e-learning and then videocall.

**Conclusion:**

Generally, respondents reported feeling neutral or confident with respect to their confidence in recognising, diagnosing, and working with patients with autism. The number of staff that have indicated interest at becoming Autism champions is a testament to the growing interest and increasing awareness about Autism.

Regarding learning disability, respondents generally reported feeling neutral or confident across the three areas of recognising moderate to severe learning disability, recognising mild learning disability, and managing/treating mental health problems in service users with learning disability.

The very high number of staff (89%) that have heard of the term “reasonable adjustments” is quite commendable and is useful to know when planning what level to ‘pitch’ training in this area.

It is interesting however that staff feel more confident at making reasonable adjustments for people with Autism, rather than for LD. One wonders whether it is due to the increasing media publicity about Autism.

